# *Calotropis procera* Latex Extract Affords Protection against Inflammation and Oxidative Stress in Freund's Complete Adjuvant-Induced Monoarthritis in Rats

**DOI:** 10.1155/2007/47523

**Published:** 2007-03-19

**Authors:** Vijay L. Kumar, Sanjeev Roy

**Affiliations:** Department of Pharmacology, All India Institute of Medical Sciences, Ansari Nagar, New Delhi 110 029, India

## Abstract

In view of the well-established anti-inflammatory properties of latex of *Calotropis procera* (DL), the present study was carried out to evaluate the protective effect of its methanol extract (MeDL) against inflammation and oxidative stress in monoarthritis induced by Freund's complete adjuvant (FCA) in rats. Intra-articular injection of FCA produced inflammation of the joint with a peak effect occurring on day 4 where a maximum increase in the levels of myeloperoxidase and inflammatory mediators like PGE_2_, TNF-*α*, and nitric oxide was observed. This was associated with oxidative stress with a marked reduction in the levels of glutathione, catalase, superoxide dismutase and glutathione peroxidase and an increase in the lipid peroxidation as indicated by the higher levels of thiobarbituric acid reactive substances (TBARSs). Subsequently on day 28 the histological analysis of the joint also revealed arthritic changes. Daily treatment of rats with MeDL (50 and 500 mg/kg) and standard anti-inflammatory drug rofecoxib (20 and 100 mg/kg), produced a significant attenuation in the inflammatory response and ameliorated the arthritic changes in the joint. The protection afforded by MeDL and rofecoxib was more pronounced than that of phenylbutazone and was associated with normalization of the levels of inflammatory mediators and biochemical parameters of oxidative stress. However, the overall protection afforded by rofecoxib was better than that of MeDL.

## 1. INTRODUCTION

The incidence of degenerative and inflammatory joint diseases, namely osteoarthritis and rheumatoid arthritis, is very high over the world [1, 2]. Typically arthritis is a common inflammatory disorder of the joint characterized by inflammation of the synovial membrane, pain, and restricted joint movement. Experimentally arthritis could be induced by various inflammagens of which Freund's complete adjuvant (FCA) is the most commonly used agent [3, 4]. Intra-articular injection of FCA is known to induce inflammation as well as immune response and to produce features that resemble rheumatoid arthritis in humans. The acute inflammatory response induced by FCA is associated with leukocyte infiltration, mast cell activation, and release of cytokines and free radicals [5, 6]. This process gets aggravated with macrophage activation and secretion of bioactive products that play an important role in tissue destruction, vascular proliferation, and fibrosis over a period of time [7].

The role of cytokines like IL-1, IL-6, tumor necrosis factor-*α* (TNF-*α*), prostaglandins (PGs), and nitric oxide (NO) in arthritis has been well established. The levels of these inflammatory mediators have been reported to be high in both experimental models of arthritis and in patients suffering from arthritis [8, 9]. Besides, generation of reactive oxygen species (ROS) and other free radicals also contribute to the pathogenesis of arthritis [10]. In view of the underlying mechanisms, both nonsteroidal and steroidal anti-inflammatory drugs are used for the management of arthritis [11]. However, due to side effects associated with the long-term use of these agents, many patients tend to use alternative therapeutic approaches including herbal therapies that have been considered safe and effective in alleviating chronic pain associated with arthritis [12].

*Calotropis procera* (Ait.) R. Br., a wild growing plant of family *Asclepiadaceae*, is well known for its medicinal properties. Different parts of this plant have been reported to exhibit anti-inflammatory, analgesic, and antioxidant properties [13]. The latex of this plant produces potent anti-inflammatory, analgesic, and weak antipyretic effects in various animal models [14–16]. Both latex and its methanol extract (MeDL) have been shown to inhibit inflammatory cell influx and edema formation induced by various inflammagens [17]. It also improves locomotor functions in experimentally induced monoarthritis in rats (unpublished findings). In view of these properties, the present study was carried out to evaluate the effect of MeDL on the levels of PGE_2_, TNF-*α*, nitric oxide (NO), myeloperoxidase (MPO), oxidative stress parameters, and joint histology in FCA-induced monoarthritis in rats. The effect of MeDL was compared with rofecoxib, a selective COX-2 (cyclooxygenase-2) inhibitor, and phenylbutazone (PBZ) a nonselective COX inhibitor.

## 2. MATERIALS AND METHODS

### 2.1. Plant material and drugs

The *C. procera* plant was identified by the Raw Materials, Herbarium and Museum Division, National Institute of Science and Communication, CSIR, New Delhi, where a voucher specimen is preserved (Voucher no. PID 1739). The latex was collected from the aerial parts of the plant growing in the wild. It was dried under shade at ambient temperature and was soxhlated to obtain methanol extract (MeDL) [18]. The MeDL was triturated with gum acacia used as suspending agent (1 : 1) in normal saline (NS), and administered orally to rats at doses ranging from 50 to 500 mg/kg (MeDL 50 and MeDL 500). Rofecoxib was administered orally at 20 and 100 mg/kg doses (Rofe 20 and Rofe 100) and phenylbutazone at a dose of 100 mg/kg (PBZ). The drugs used in the study were obtained from Arbro Pharmaceuticals (New Delhi, India) (rofecoxib and phenylbutazone). Freund's complete adjuvant was obtained from Sigma-Aldrich Corporation (Bangalore, India).

### 2.2. Animals

The study was carried out on 5-6-month-old Wistar rats of either sex weighing 150–180 g. The rats were obtained from the Experimetal Animal Facility of the Institute, were kept at ambient temperature, and had free access to water and diet. The animal experiments were carried in accordance with the guidelines of Institutional Animal Ethics Committee.

### 2.3. Experimental design

Monoarticular arthritis was induced in rats by injecting 0.1 mL of 0.1% FCA (Sigma Aldrich, USA) into the intra-articular space of right ankle joint (day 0) [19]. The increase in joint diameter was measured daily starting from day 0, using a screw gauge till the time of peak inflammation (day 4), and then it was measured every fourth day for a period of 28 days. The rats were divided into seven groups, consisting of six animals each for analysis of histological and biochemical parameters. Group I: normal control; Group II: FCA control. In Group III to Group VII, drugs were administered orally as suspension with gum acacia in NS, 1 hour before injecting FCA on day 0 and then daily either for 4 days or for 28 days at doses based on our earlier studies where no observable toxic effects were seen [17, 20, 21], Group III: MeDL (50 mg/kg, MeDL 50); Group IV: MeDL (500 mg/kg, MeDL 500); Group V: rofecoxib (20 mg/kg, Rofe 20); Group VI: rofecoxib (100 mg/kg, Rofe 100); Group VII: phenylbutazone (100 mg/kg, PBZ).

### 2.4. Determination of levels of oxidative stress parameters and inflammatory mediators

The levels of biochemical markers of oxidative stress and inflammatory mediators were determined at the site of inflammation. Animals were sacrificed at the time of peak inflammation (day 4) and the tissue of the arthritic joint was removed and processed for the estimation of glutathione (GSH, mg/g tissue) [22], catalase (U/mg protein) [23], superoxide dismutase (SOD, U/mg protein) [24], glutathione peroxidase (GPx, U/mg protein) [25], thiobarbituric acid-reactive substances (TBARSs) as a measure of malondialdehyde (MDA, nmol/g tissue) [26], nitric oxide (NO, *μ*M/mg tissue) [27], prostaglandin E_2_ (PGE_2_, pg/mg tissue, R&D Systems), tumor necrosis factor-*α* (TNF-*α*, pg/mg tissue, Diaclone Research), and myeloperoxidase (MPO, OD/mg tissue) [28] levels.

### 2.5. Estimation of protein

The protein concentration of the samples was determined by Bradford's method [29].

### 2.6. Histological analysis

Rats were sacrificed on day 28, the limbs were removed above the stifle joints, degloved and fixed in 1% formaldehyde in saline. They were decalcified in EDTA, processed for paraffin embedding, sectioned, and stained with hematoxylin-eosin [30]. The sections were examined for arthritic changes in the control as well as in the drug-treated rats.

### 2.7. Statistical analysis

The values are expressed as mean ± SEM of six observations and ANOVA was used to compare the groups. The statistical analysis was carried out by the version 10 of the SPSS program and the values of *P* < .05 were considered as statistically significant.

## 3. RESULTS

### 3.1. Effect of MeDL on joint inflammation

**Figure 1:**
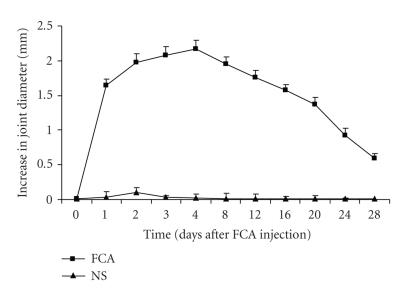
Time course for increase in joint diameter in FCA-induced monoarthritis in rats. Values are mean ± SEM.

Injection of FCA into right ankle joint of rat produced an increase in joint diameter that was maximum on day 4 (2.17 ± 0.13 mm), and thereafter it gradually declined. Injection of NS on the other hand produced a marginal increase in the joint diameter on day 2 (0.04 ± 0.10 mm) that returned to normal within 4 days (Figure 1).

The inhibitory effect of various drugs was evaluated on the day of peak inflammation, that is, day 4. Oral administration of MeDL produced a dose-dependent decrease in joint inflammation and the increase in joint diameter was 1.59 ± 0.09 mm and 1.20 ± 0.08 mm in MeDL 50 and MeDL 500 groups against 2.17 ± 0.13 mm in FCA control (27% and 45% inhibition). COX-2 selective inhibitor, rofecoxib, was more effective in inhibiting joint inflammation as compared to MeDL. The increase in joint diameter in Rofe 20 and Rofe 100 groups was 1.66 ± 0.08 mm and 0.70 ± 0.33 mm (24% and 68% inhibition). PBZ, a nonselective COX inhibitor produced 16% inhibition in joint inflammation with the increase in joint diameter of 1.82 ± 0.12 mm (Table 1).

### 3.2. Effect of MeDL on tissue levels of inflammatory mediators

**Figure 2:**
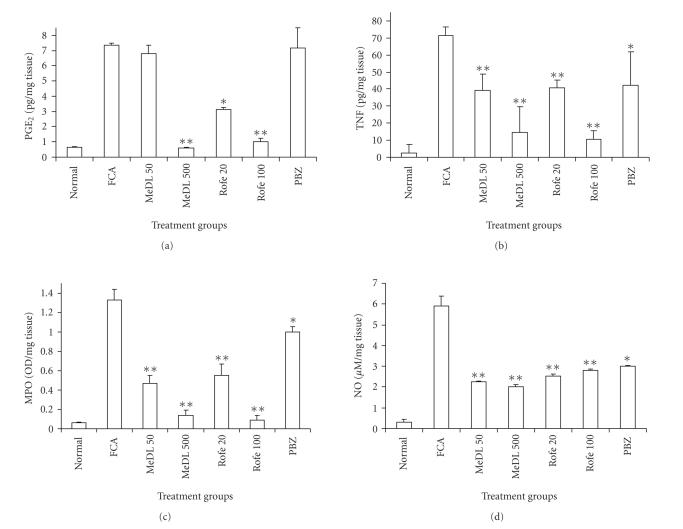
Effect of drugs on the tissue level of PGE_2_, TNF-*α*, MPO, and NO in FCA-induced monoarthritis in rats. Values are mean ± SEM. *P < 0.05, **P < 0.001.

The inflammation induced by FCA was associated with an increase in the levels of PGE_2_ and TNF-*α*. The tissue levels of PGE_2_ and TNF-*α* were 7.35 ± 0.14 and 71.5 ± 5.00 pg/mg tissue in the FCA control as compared to 1.00 ± 0.01 and 2.50 ± 5.00 pg/mg tissue in normal control rats, respectively. Both MeDL and rofecoxib produced a significant decrease in the levels of PGE_2_ and TNF-*α* (*P* < .005). The levels of PGE_2_ in MeDL 500 group were 0.6 ± 0.05, and in Rofe 100 group were 1.00 ± 0.23, and that of TNF-*α* in MeDL 500 group were 14.50 ± 15.00, and in Rofe 100 group were 10.50 ± 5.00 pg/mg tissue, respectively. PBZ on the other hand was not effective in reducing the tissue PGE_2_ levels and was only marginally effective in reducing the tissue TNF-*α* levels (Figure 2). FCA injection produced a significant increase in tissue MPO activity from 0.06 ± 0.01 OD/mg tissue in normal control rats to 1.33 ± 0.11 OD/mg tissue. Treatment with MeDL and rofecoxib significantly reduced the tissue MPO activity and their effect was comparable in this regard. The MPO levels were 0.14 ± 0.02 and 0.09 ± 0 OD/mg tissue in MeDL 500 and Rofe 100 group, respectively. PBZ on the other hand was marginally effective in decreasing the MPO levels as compared to FCA control (1.00 ± 0.03 versus 1.33 ± 0.11 OD/mg tissue) (Figure 2). MeDL and rofecoxib were also equieffective in reducing the tissue NO levels in the arthritic rats (2.0 ± 0.11 and 2.8 ± 0.10 against 5.9 ± 0.50 *μ*M/mg tissue in FCA control). The effect of PBZ in this regard was comparable to that of MeDL and rofecoxib (3.0 ± 0.04 *μ*M/mg tissue) (Figure 2).

### 3.3. Effect of MeDL on tissue levels of GSH, catalase, SOD, GPx, and TBARS

Oxidative stress associated with FCA-induced monoarthritis was evaluated by measuring the levels of GSH, catalase, SOD, GPx, and TBARS in the inflamed joint tissue. FCA injection into the ankle joint markedly decreased the tissue GSH, catalase, SOD, and GPx levels from 18.20 ± 1.10 mg/g tissue, 28.60 ± 0.15 U/mg protein, 277.70 ± 0.15 U/mg protein, and 31.40 ± 0.10 U/mg protein in normal control rats to 4.80 ± 0.40 mg/g tissue, 0.17 ± 0.02 U/mg protein, 79.90 ± 0.10 U/mg protein, and 5.97 ± 0.05 U/mg protein, respectively. Both MeDL and rofecoxib produced a dose-dependent increase in the level of these oxidative stress parameters. On the other hand, FCA produced a marked increase in the levels of TBARS from 3.50 ± 0.50 nmol/g tissue to 103.00 ± 3.00 nmol/g tissue. Both MeDL and rofecoxib produced a dose-dependent decrease in the levels of TBARS and the effect of these drugs was comparable. PBZ, on the other hand, produced a marginal change in the levels of all the oxidative stress parameters as compared to FCA control (Table 2).

### 3.4. Effect of MeDL on joint histology

**Figure 3:**
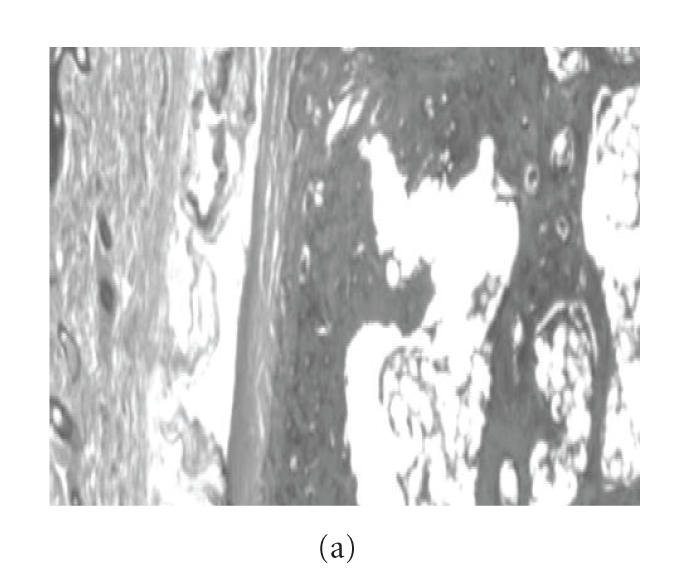

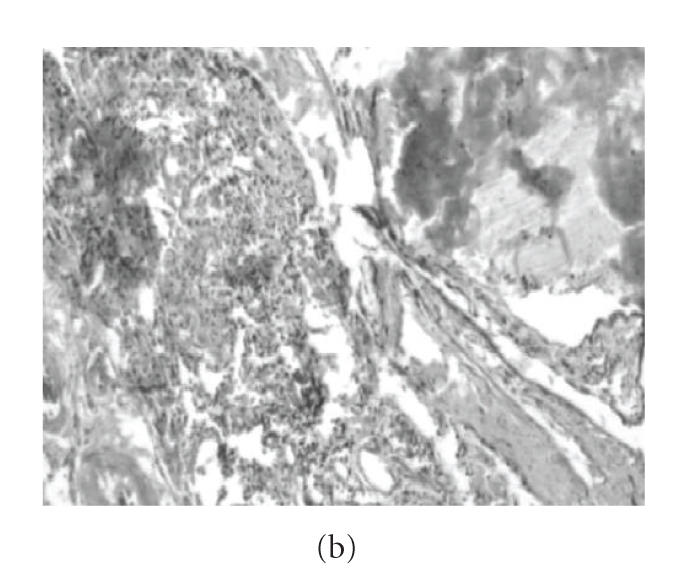

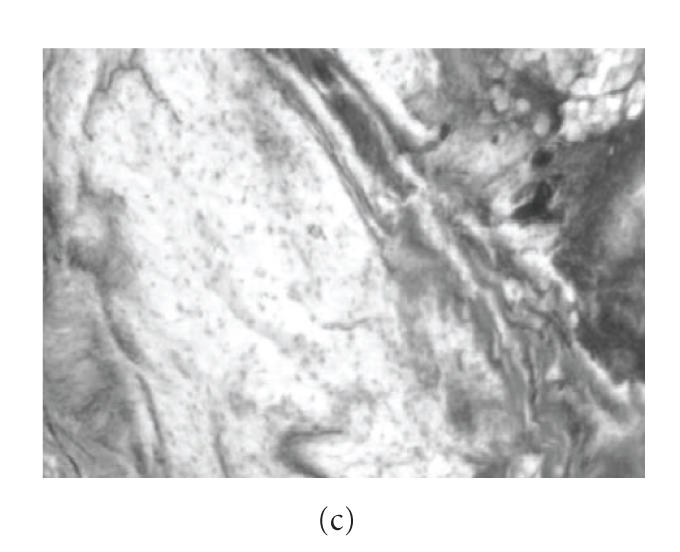

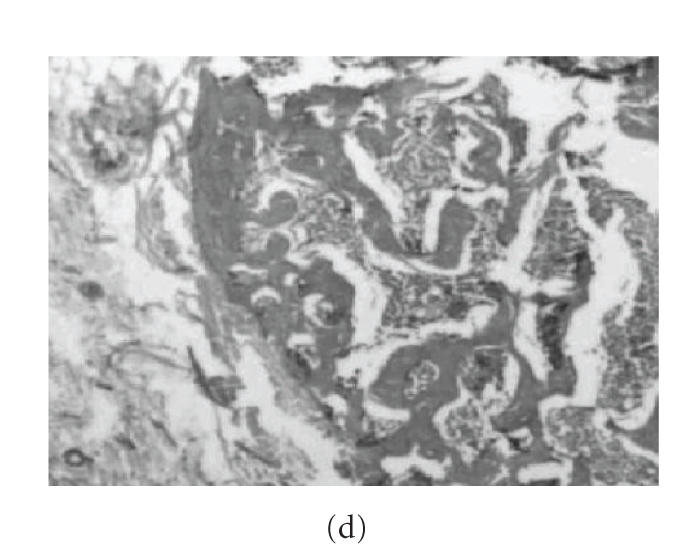
Effect of drugs against FCA-induced arthritic changes as revealed by histological analysis: (a) normal control; (b) FCA control; (c) MeDL 500 mg/kg; (d) Rofe 100 mg/kg.

The inflammation induced by FCA was associated with cellular infiltration, edema, granuloma formation, and bone destruction on day 28 (Figure 3(b)).

Both MeDL 500 and Rofe 100 significantly decreased the arthritic changes as compared to FCA control, however, rofecoxib was more effective in this regard (Figures 3(c) and 3(d)).

## 4. DISCUSSION

The latex of *Calotropis procera* is well known for its anti-inflammatory properties in various experimental models. It has also been shown to afford protection against functional impairment produced by FCA in rat model of monoarthritis. In the present study, we have evaluated the effect of latex of *C. procera* on the levels of inflammatory mediators, oxidative stress parameters, and joint histology in FCA-induced monoarthritis model and compared it with rofecoxib. Intra-articular injection of FCA produced a peak inflammatory response in the joint on day 4 that is associated with fluid exudation, neutrophil infiltration, and mast cell activation [31, 32]. This was followed by a slow regression and the joint swelling continued up to day 28 possibly due to oil-based adjuvant and the antigenicity of mycobacterium [33]. The inhibitory effect of drugs was evaluated against FCA-induced inflammation on day 4. MeDL produced a dose-dependent inhibition in joint inflammation that could be attributed to its ability to inhibit cellular influx and vascular permeability [17, 20]. It has earlier been shown to inhibit inflammatory response induced by various mediators and inflammagens like histamine, bradykinin, prostaglandins, carragenin, and compound 48/80 [17]. The role of various inflammatory mediators in adjuvant-induced arthritis has been well established [34, 35]. In our study, rofecoxib, a selective COX-2 inhibitor, was found to be more effective than MeDL and phenylbutazone in inhibiting the FCA-induced joint inflammation as reported earlier by Kumar et al. [21] and Francischi et al. [36]. Rofecoxib acts by inhibiting COX-2 that plays an important role in an inflammatory response. The greater efficacy of rofecoxib could be attributed to its better distribution at the site of inflammation as suggested for other COX-2 inhibitors [37]. Further, rofecoxib was also found to be more effective as compared to MeDL in inhibiting cell influx and bone destruction as revealed by histological analysis. The inhibitory effect of MeDL and rofecoxib on cell influx was further substantiated by their ability to decrease tissue MPO activity that has been used as an index of granulocyte infiltration. It is interesting to note that PBZ produced only a marginal decrease in tissue MPO activity. The inability of PBZ to inhibit cellular influx has also been reported by Meacock and Kitchen [38] and Arya and Kumar [17].

The neutrophilic recruitment at the site of inflammation has been reported to involve TNF-*α* production that induces the synthesis of LTB4, a well-known chemoattractant and prostaglandins that plays a key role in the pathogenesis of inflammatory diseases. Elevated levels of TNF-*α* and prostaglandins have been reported in arthritic patients and in experimentally induced arthritis [39, 40]. In our study, both MeDL and rofecoxib produced a marked reduction in the tissue levels of TNF-*α* and PGE_2_. However, PBZ was ineffective in reducing the levels of PGE_2_ though it produced a significant decrease in tissue TNF-*α* levels. A marked reduction in the levels of PGE_2_ brought about by MeDL was comparable to that of rofecoxib and suggests that like rofecoxib, MeDL might be inhibiting COX-2. Earlier, the MeDL was shown to inhibit inflammation induced by PGE_2_ [17].

The role of NO has been well established in an inflammatory response. As the inflammatory response progresses, large quantities of NO are generated through the induction of iNOS (inducible nitric oxide synthase) that reacts with superoxide anion to form peroxynitrate, a potent oxidizing molecule capable of eliciting lipid peroxidation. Lipid peroxidation is the oxidative deterioration of polyunsaturated lipids to form radical intermediates that bring about cellular damage. MDA, a major end product of this reaction, is an index of lipid peroxidation and has been estimated as TBARS [41]. In our study, both MeDL and rofecoxib brought down the tissue levels of NO and TBARS. Besides, the infiltrating cells also generate reactive oxygen species and free radicals that bring about destruction of the inflamed joint. As a result, the scavenging enzyme SOD that leads to the formation of hydrogen peroxide is utilized and its activity is reduced in arthritic rats. The hydrogen peroxide thus generated is decomposed by catalase and glutathione peroxidase. Excessive production of lipid hydroperoxide may also contribute to decreased activity of GPx in arthritic condition [42]. Beside enzymatic antioxidants, the level of glutathione, a nonenzymatic reducing agent that traps free radicals and prevents oxidative stress, is also decreased in arthritis [43]. Both MeDL and rofecoxib maintained the oxidative homeostasis, and the levels of GSH and activities of catalase, SOD, and GPx were comparable to the control animals. The antioxidant properties of rofecoxib and latex of *C. procera* have also been reported earlier [44, 45].

Thus, present study shows that the latex of *C. procera* markedly reduces cell influx, release of mediators, and oxidative stress associated with arthritic condition, and therefore has the potential to be used as an antiarthritic agent.

## Figures and Tables

**Table 1:** Inhibition of joint inflammation by various drugs in FCA-induced monoarthritis. Values given are mean ± SEM (*n* = 6).

Treatment groups	Dose (mg/kg)	Increase in joint diameter (mm)	Inhibition (%)

Normal control	—	—	—
FCA control	—	2.17 ± 0.13	—
MeDL	50	1.59 ± 0.09	27%
MeDL	500	1.20 ± 0.08*	45%
Rofecoxib	20	1.66 ± 0.08*	24%
Rofecoxib	100	0.70 ± 0.33*	68%
Phenylbutazone	100	1.82 ± 0.12	16%

*P < .05.

**Table 2:** Effect of drugs on parameters of oxidative stress in FCA-induced monoarthritis. Values given are mean ± standard error of the mean (*n* = 6).

Groups	GSH	Catalase	SOD	GPx	TBARS
	(mg/g tissue)	(U/mg protein)	(U/mg protein)	(U/mg protein)	(nmol/g tissue)

Normal control	18.20 ± 1.10	28.60 ± 0.15	277.70 ± 0.15	31.40 ± 0.10	3.50 ± 0.50
FCA control	4.80 ± 0.40	0.17 ± 0.02	79.90 ± 0.10	5.97 ± 0.05	103.00 ± 3.00
MeDL 50	7.30 ± 0.40*	0.21 ± 0.06	95.70 ± 0.08	9.61 ± 0.03*	77.50 ± 6.50
MeDL 500	11.30 ± 0.50**	20.10 ± 0.01**	222.11 ± 0.02**	29.52 ± 0.11**	5.00 ± 1.00**
Rofecoxib 20	6.80 ± 1.00	6.56 ± 0.01*	137.16 ± 0.03	6.55 ± 0.08	64.00 ± 7.00*
Rofecoxib 100	14.30 ± 0.90**	22.40 ± 0.02**	236.62 ± 0.10**	28.16 ± 0.01**	5.50 ± 0.50**
PBZ	7.20 ± 0.80*	4.16 ± 0.06*	94.62 ± 0.02	6.52 ± 0.01	86.5 ± 5.5

*P < .05.

**P < .001.
